# Supplementary material for: Essential role of the Crk family-dosage in DiGeorge-like anomaly and metabolic homeostasis
Source: Life Sci Alliance. 2020 Feb 10;3(2):e201900635. doi: 10.26508/lsa.201900635 (PMC7010317; doi:10.26508/lsa.201900635)
Supplement: Supplementary file 1 [file LSA-2019-00635_TableS1.doc]

Table S1 Genetic Interaction between *Tbx1* and *Crk* in mice

| Genotype | Abnormal (Total) | aortic arch defects | | Thymic hypoplasia | Dermal vascular defects |
| --- | --- | --- | --- | --- | --- |
|  |  | Ab-RSA | IAAB (with RAA or CAA) |  |  |
| wild type | 0 (19) | 0 | 0 | 0 | 0 |
| *Tbx1^-^*/+ | 5 (22) | 4 | 1 (0) | 0 | 0 |
| *Crk^d^*/+ | 0 (17) | 0 | 0 (0) | 0 | 0 |
| *Crk^d^*/+; *Tbx1^-^*/+ | 15* (18) | 10** | 6^#^ (3) | 6^##^ | 3 |
|  | | | | | |

Embryos were isolated at E16.5 upon timed mating between *Crk ^d^*^/+^ and *Tbx1 -*^/+^ parents or between *Crk ^d^*^/+^; *Tbx1^-^*^/+^ and C57BL/6J parents. Ab-RSA, abnormal origin of right subclavian artery; IAAB, interrupted arch of aorta type B; RAA, right-sided arch of aorta; CAA, cervical arch of aorta. Dermal vascular defects were enlargement of dermal vessels associated with small hemorrhagic regions. Note that whereas IAAB without RAA or CAA would not be viable after birth, IAAB with RAA or CAA would likely survive. We recovered some compound heterozygous *Crk ^d^*^/+^; *Tbx1 -*^/+^ mice that were viable and fertile.

* *p.adj* = 5.2e-07, 0.00066, and 8.8e-07 when compared to wild type, *Tbx1^-^*/+, and *Crk^d^/+* groups, respectively, by Fisher’s exact test with FDR adjustments for multiple pairwise comparisons (Benjamini and Hochberg). FDR adjustments provide stricter statistical evaluations than simple pairwise comparisons with Fisher’s exact test alone. The *Tbx1^-^*/+ group with 5 abnormal embryos had a *p.adj* value of 0.30 or 0.34, compared to the wild type or *Crk ^d^*^/+^
groups, respectively. Statistical calculations were performed using the *R* package *RVAideMemoire*.

** *p.adj* = 0.00075, 0.042, and 0.0010 compared to wild type, *Tbx1^-^*/+ and *Crk^d^/+* groups, respectively, as calculated above.

^#^ *p.adj* = 0.048, 0.066, and 0.057 compared to wild type, *Tbx1^-^*/+ and *Crk^d^/+* groups, respectively, as calculated above, thus indicating that the IAAB frequency in *Crk^d^*/+; *Tbx1^-^*/+ was only marginal compared to the other genotypes due to a very rare case of IAAB in the *Tbx1^-^*/+ group in this study. In our previous report (Guris et al., 2006), no IAAB case was observed among 18 *Tbx1^-^*/+ embryos at E16.5, whereas 4 cases of Ab-RSA were found in the same group.

^##^ *p.adj* = 0.024, 0.024, and 0.038 compared to wild type, *Tbx1^-^*/+ and *Crk^d^/+* groups, respectively, as calculated above.
