# Supplementary material for: Essential role of the Crk family-dosage in DiGeorge-like anomaly and metabolic homeostasis
Source: Life Sci Alliance. 2020 Feb 10;3(2):e201900635. doi: 10.26508/lsa.201900635 (PMC7010317; doi:10.26508/lsa.201900635)
Supplement: Supplementary file 3 [file LSA-2019-00635_TableS2.doc]

Table S2. RNA-Seq read depths and mapping efficiency.

| Genotype* | Sample | Total read count | Aligned read count | Efficiency (%) |
| --- | --- | --- | --- | --- |
| Wildtype | primary_281OHT | 24,924,467 | 23,488,145 | 94.24 |
|  | primary_281 | 41,398,970 | 39,083,927 | 94.41 |
|  | primary_282OHT | 34,250,552 | 32,271,015 | 94.22 |
|  | primary_282 | 30,929,202 | 29,103,526 | 94.10 |
| *Crk*^f/f^ | primary_258OHT | 26,491,776 | 24,974,515 | 94.27 |
|  | primary_258 | 26,074,615 | 24,630,142 | 94.46 |
|  | primary_284OHT | 24,636,608 | 23,039,189 | 93.52 |
|  | primary_284 | 28,251,841 | 26,583,808 | 94.10 |
|  | primary_285OHT | 27,638,871 | 25,865,027 | 93.58 |
|  | primary_285 | 25,328,731 | 23,735,169 | 93.71 |
|  | primary_286OHT | 47,512,544 | 44,301,452 | 93.24 |
|  | primary_286 | 21,607,064 | 20,277,130 | 93.84 |
| *Crk*^f/f^;*Crkl*^f2/f2^ | primary_387OHT | 26,813,125 | 25,501,395 | 95.11 |
|  | primary_387 | 32,241,588 | 30,525,857 | 94.68 |
|  | primary_388OHT | 26,263,335 | 25,139,061 | 95.72 |
|  | primary_388 | 22,797,920 | 21,788,851 | 95.57 |
|  | primary_436OHT | 27,314,368 | 26,177,961 | 95.84 |
|  | primary_436 | 33,897,270 | 32,488,822 | 95.84 |
|  | primary_437OHT | 31,462,751 | 30,148,328 | 95.82 |
|  | primary_437 | 30,030,077 | 28,837,725 | 96.03 |
| *Crkl*^f2/f2^ | primary_421OHT | 25,201,415 | 23,657,212 | 93.87 |
|  | primary_421 | 29,449,573 | 27,683,914 | 94.00 |
|  | primary_424OHT | 32,836,634 | 30,802,437 | 93.81 |
|  | primary_424 | 32,089,017 | 30,131,336 | 93.90 |
|  | primary_426OHT | 26,672,234 | 24,794,808 | 92.96 |
|  | primary_426 | 30,109,351 | 28,335,546 | 94.11 |
|  | primary_427OHT | 27,861,442 | 25,897,966 | 92.95 |
|  | primary_427 | 25,889,254 | 24,126,191 | 93.19 |

*All primary MEFs listed above are heterozygous for *Rosa26^creERT2^* to trigger gene deficiency of either or both *Crk* and *Crkl* in primary mouse embryonic fibroblasts (MEFs) by 4-hydroxytamoxifen (OHT). This strategy permitted a pairwise experimental design, with or without deficiency induction, in each individual batch of primary MEFs. Each genotype group consists of a data structure from four independent batches of primary MEFs, while the Wildtype group included two batches as a negative control group (in which *Crk* or *Crkl* deficiency could be induced by OHT). Each MEF batch was cultured from a single independent embryo. For example, the data subgroup of ‘primary_427’ and ‘primary_427OHT’ was obtained from the MEF ID#427 cultured from the embryo ID#427.
