# Supplementary material for: Essential role of the Crk family-dosage in DiGeorge-like anomaly and metabolic homeostasis
Source: Life Sci Alliance. 2020 Feb 10;3(2):e201900635. doi: 10.26508/lsa.201900635 (PMC7010317; doi:10.26508/lsa.201900635)
Supplement: Supplementary file 6 [file LSA-2019-00635_TableS3.doc]

Table S3. PCR primers used in this study

^1^Matusi et al., Proviral silencing in embryonic stem cells requires the histone methyltransferase ESET. Nature 464, 927-931 (2010)

| Names (Use) | Sequences (5’ to 3’) | Locations | Chromosomal location in mm9 |
| --- | --- | --- | --- |
| Crk-4750F (genotyping) | ACCGGCCGCTACCAATCTGTCAAA | exon1 upstream |  |
| Crk-i1F1 (genotyping) | AACCTGGCGGGGGAGCTGTCTTCT | intron1 |  |
| Crk-6034R (genotyping) | ACCGAAGGCCACCAGGGGAGTAT | intron1 |  |
| Crkl-int1-F2 (genotyping) | TCAGCCAGGAGAGGAGGATCATCAA | intron1 |  |
| Crkl-int1-R (genotyping) | TGGCTCTCGTCCTGCATAGGTCA | intron1 |  |
| Crkl-int2-R1 (genotyping) | GAGGCCAGGACAGCTGGGACTAC | intron2 |  |
| Cre-1907F (genotyping) | CTGGAAAATGCTTCTGTCCGTTT | Cre |  |
| Cre-2269R (genotyping) | GCCAGATTACGTATATCCTGGCA | Cre |  |
| R26-SA-R (genotyping) | GCGAAGAGTTTGTCCTCAAC | splice acceptor |  |
| R26-F2 (genotyping) | AAAGTCGCTCTGAGTTGTTAT | Gt(ROSA)26 |  |
| R26-R (genotyping) | GGAGCGGGAGAAATGGATATG | Gt(ROSA)26 |  |
|  |  |  |  |
| Gapdh-F (ChIP-qPCR) | ATCCTGTAGGCCAGGTGATG | Ref1 |  |
| Gapdh-R (ChIP-qPCR) | AGGCTCAAGGGCTTTTAAGG |  |  |
| Pfkl-F (ChIP-qPCR) | ATCCATCCCTAAACACGCCC | exon1-intron1 | chr10:77472310+77472384 |
| Pfkl-R (ChIP-qPCR) | CAAGGCCATTGGAGTGCTGA |  |  |
| Pgk1-F (ChIP-qPCR) | GCGGGTCGTGATGAGGTAAT | exon1-intron1 | chrX:103382640+103382728 |
| Pgk1-R (ChIP-qPCR) | GATGGGAAAACCGTGCTTGC |  |  |
| Eno1-F (ChIP-qPCR) | CTATTGGAGTGATGGCACGC | 5'UTR-exon1 | chr4:149611229+149611334 |
| Eno1-R (ChIP-qPCR) | CTGTCTTCCTTTCCTGCTCGT |  |  |
| Ldha-F (ChIP-qPCR) | CTGGGCTCCCACTCTGAC | 5'UTR-exon1 | chr7:54101148+54101229 |
| Ldha-R (ChIP-qPCR) | GACAGTGGCTCCAGCACG |  |  |
| Pgam1-F (ChIP-qPCR) | TTAGCAGGCCGCAGAACTTG | 5'UTR-exon1 | chr19:41986336-41986416 |
| Pgam1-R (ChIP-qPCR) | TCTTTCCACTCGCTCGACAG |  |  |
|  |  |  |  |
| Gapdh-F (RT-qPCR) | ATGGTGAAGGTCGGTGTGAACGGATTTGGC | exons 2-3 |  |
| Gapdh-R (RT-qPCR) | AGCTTCCCATTCTCGGCCTTGACTGTGCCGT | exon3 |  |
| Pfkl-F (RT-qPCR) | TGGCAGACTATGTGCTGGGGAGC | Ref2 |  |
| Pfkl-R (RT-qPCR) | GCTAGCACTGGGAGGGTGAGAGTC |  |  |
| Pgk1-F (RT-qPCR) | AAGTCCTTCCTGGGGTGGTGCTC | Ref2 |  |
| Pgk1-R (RT-qPCR) | AGGGTTCCTGGTGCCACATCTCAG |  |  |
| Eno1-F (RT-qPCR) | TAGGCATCCACACCTGACCACCAG | Ref2 |  |
| Eno1-R (RT-qPCR) | GGGCTCCAGACACTAGCGGGAAG |  |  |
| Ldha-F (RT-qPCR) | CACTGACTCCTGAGGAAGAGGCCC | Ref2 |  |
| Ldha-R (RT-qPCR) | AGCTCAGACGAGAAGGGTGTGGTC |  |  |
| Pgam1-F (RT-qPCR) | GTTCTCGGACCACATCGAGGGACA | Ref2 |  |
| Pgam1-R (RT-qPCR) | TGCAGGACAGGTTCCAGGGACAAA |  |  |
| Igf1-F-set1 (RT-qPCR) | GTGTGGACCGAGGGGCTTTTACTTC | Ref3 |  |
| Igf1-R-set1 (RT-qPCR) | GCTTCAGTGGGGCACAGTACATCTC |  |  |
| Igf1-F-set2 (RT-qPCR) | TGCTTGCTCACCTTCACCA | Ref4 |  |
| Igf1-R-set2 (RT-qPCR) | CAACACTCATCCACAATGCC |  |  |

^2^Baba et al., Glycolytic genes are targets of the nuclear receptor Ad4BP/SF-1. Nat Commun 5, 3634 (2014)

^3^Sl et al., Adult-onset degeneration of adipose tissue in mice deficient for the Sox8 transcription factor. J Lipid Res 50, 1269-1280 (2009)

^4^Carroll et al., Expression of a pathogenic mutation of SOD1 sensitizes aprataxin-deficient cells and mice to oxidative stress and triggers hallmarks of premature ageing. Hum Mol Genet 24, 828-840 (2015)
